# Long non-coding RNAs in hematologic malignancies: road to translational research

**DOI:** 10.3389/fgene.2013.00250

**Published:** 2013-11-20

**Authors:** Mohammadreza Hajjari, Atefeh Khoshnevisan, Young Kee Shin

**Affiliations:** ^1^Department of Genetics, Shahid Chamran University of AhvazAhvaz, Iran; ^2^Department of Genetics, School of Biological Sciences, Tarbiat Modares UniversityTehran, Iran; ^3^Laboaratory of Molecular Pathology and Cancer Genomics, Department of Pharmacy, Seoul National University College of PharmacySeoul, Korea

**Keywords:** long non-coding RNA (lncRNA), expression, translational research, leukemia, carcinogenesis

The human genome encodes ~20,000 proteins; however, protein-coding genes represent <2% of the total genome (Gibb et al., 2011). Since this discovery, several studies have pointed out that at least 90% of the genome is actively transcribed (Birney et al., 2007; Costa, 2010). This field provoked a spirited debate, which is evident from the number of original articles, reviews, letter to the editors, commentaries, and rebuttals.

Many long non-coding RNAs (lncRNAs), ranging from 0.2 to ~100 kilobases (kb) in length, are transcribed from the genome. LncRNAs affect various cellular functions such as gene regulation, genomic imprinting, RNA maturation, and translation (Wang and Chang, 2011). In addition, the differential expression of lncRNAs has recently been linked to carcinogenesis, including gastric cancer (Hajjari et al., 2013). In recent reports, lncRNAs have been attributed to oncogenic and/or tumor suppressor roles (Reviewed in Gibb et al., 2011; Qiu et al., 2013). Taken together, these reports support the possible involvement of lncRNAs in the initiation and/or progression of breast, colorectal, liver, lung, and gastric cancers (Hajjari and Khoshnevisan, 2013). However, little is known about the potential role of lncRNAs in leukemia. In fact, there is a paucity of reports on the characterization of lncRNAs in this type of cancer.

In the December 2011 edition of *Frontiers in Genetics*, Heuston et al. (2011) published a review on non-coding RNAs in hematologic malignancies. They highlighted the potential roles for non-coding RNAs including microRNAs and lncRNAs in the development of acute and chronic leukemia. While this is the only review that described the expression patterns of ncRNAs in leukemia, it may provide a foundation for future research in this field.

Heuston et al., referred to several reports on lncRNAs such as *ANRIL, lncRNA-P21, MEG3, Dleu2, HOTAIRM1, EGO*, and *lncRNA-a7* in leukemia, highlighting the potential benefit of research on lncRNAs for the development of much needed diagnostic, prognostic, and therapeutic targets (Heuston et al., 2011). In addition, based on the previous studies, Heuston et al., proposed to study more about the functional role of *MEG3* in leukemia. In our opinion, this review provides an overview of the published reports on the subject and represents a suitable guide for researchers involved in the field of molecular pathology of leukemia.

To test if any prior evidence on the potential role of lncRNAs in leukemia progression existed, we queried the Oncomine Database (www.oncomine.org) for five lncRNAs: *HOTAIR, ANRIL, MEG3, H19,* and *UCA1*. All five lncRNAs were among the most cited lncRNAs associated with a potential role in cancer.

We found that two lncRNAs including *MEG3* and *H19* are up-regulated in acute myeloid leukemia (AML) cancer compared to normal blood cells (Fold change > 1.5, *P*-value < 0.01). As presented in Table 1, the results showed that *H19, UCA1, and MEG3* are up-regulated in French-American-British (FAB) classification subtypes M3, M2, and M3, respectively. The expression of *H19* is up-regulated in AML compared to acute lymphoblastic leukemia (ALL). In conclusion, taken together, the review from Heuston et al. and our data shed lights on the roles of lncRNAs in different types of leukemia and may prove useful for the development of molecular diagnosis and targeted therapy for leukemia.

**Table 1:** **Expression level of the lncRNAs in Leukemia (the results are expressed as fold change between cancer and normal tissues or cancer subtypes)**.

**lncRNA**	**State 1**	**State 2**	**Fold**	***P*-value**
	**(Up-regulated)**	**(Down-regulated)**	**change**	
*MEG3*	AML	Normal	3.592	0.004
	FAB M3	Other FAB subtypes	14.617	1.23 × 10^−7^
*H19*	AML	Normal	1.932	5.4 × 10^−4^
	FAB M3	Other FAB subtypes	4.271	2.26 × 10^−4^
	AML	ALL	2.664	2 × 10^−6^
*UCA1*	FAB M2	Other FAB subtypes	1.885	3.33 × 10^−4^

The data were obtained from the Oncomine Database.
